# NPR-C gene polymorphism is associated with increased susceptibility to coronary artery disease in Chinese Han population: a multicenter study

**DOI:** 10.18632/oncotarget.9358

**Published:** 2016-05-13

**Authors:** Qin Hu, Qiji Liu, Shasha Wang, Xi Zhen, Zhimian Zhang, Ruijuan Lv, Guihua Jiang, Zhiyong Ma, Hong He, Daqing Li, Xiaoling Liu, Fei Gao, Jifu Li, Li Li, Mei Zhang, Xiaoping Ji, Yuguo Chen, Daowen Wang, Dejia Huang, Aiqun Ma, Wei Huang, Yuxia Zhao, Yaoqin Gong, Cheng Zhang, Yun Zhang

**Affiliations:** ^1^ Key Laboratory of Cardiovascular Remodeling and Function Research, Chinese Ministry of Education and Chinese Ministry of Health, Department of Cardiology, Shandong University Qilu Hospital, Jinan, Shandong, China; ^2^ Department of Medical Genetics, School of Medicine, Key Laboratory of Experimental Teratology, Ministry of Education, Shandong University, Jinan, Shandong, China; ^3^ Medical Examination Center, Shandong University Qilu Hospital, Jinan, Shandong, China; ^4^ Department of Emergency, Shandong University Qilu Hospital, Jinan, Shandong, China; ^5^ Department of Internal Medicine, Tongji Hospital, Tongji Medical College, Huazhong University of Science & Technology, Wuhan, China; ^6^ Division of Cardiology, Department of Medicine, West China Hospital, Sichuan University, Chengdu, Sichuan, China; ^7^ Department of Cardiology and Periphery Vascular Medicine, The First Affiliated Hospital of Medical College of Xi'an Jiaotong University, Xi'an, Shaanxi, China; ^8^ Ruijin Hospital, School of Medicine, Shanghai Jiaotong University, Department of Genetics, Chinese National Human Genome Center, Shanghai, China

**Keywords:** natriuretic peptide receptor C, coronary artery disease, genome-wide association studies, single nucleotide polymorphisms, susceptibility gene, Pathology Section

## Abstract

To find a new locus that confers significant susceptibility to CAD in Chinese Han population, a genome-wide association study in 200 “extreme individuals” from a Shandong cohort and a pathway-based candidate gene study from a Shanghai cohort (293 CAD/293 controls) were simultaneously performed. Amongst them, 13 SNPs associated with CAD were selected to conduct validation and replication studies in additional 3363 CAD patients and 3148 controls. A novel locus rs700926 in natriuretic peptide receptor C (NPR-C) was identified in Shandong and Hubei cohorts. Then rs700926 and other nine tag SNPs were genotyped in four geographically different populations (Shandong, Shaanxi, Hubei and Sichuan cohorts), and 6 SNPs (rs700926, rs1833529, rs2270915, rs17541471, rs3792758 and rs696831) showed stronger association with CAD, regardless of single or combined analysis. We further genotyped rs2270915 and 10 additional tag SNPs in a central China cohort and identified rs12697273 and rs10066436 as the loci associated with CAD. All these positive associations remained significant after adjustment for traditional risk factors of CAD. NPR-C gene SNPs significantly contribute to CAD susceptibility in the Chinese Han population.

## INTRODUCTION

Coronary artery disease (CAD) is the leading cause of mortality worldwide and more than 700,000 people die from CAD each year in China [1]. Current evidence suggests that CAD is a heritable trait activated by a number of biological pathways and responsive to environmental stimuli [2] Recently, genome-wide association studies (GWAS), a technology of chip array using hundreds of thousands of markers and targeted gene-based resequencing, have facilitated the gene discovery for CAD. Previous GWAS in populations of European ancestry identified several genetic loci on chromosomes 1p13, 1q41, 2q36, 3q22, 6p24, 6q25, 9p21, 10q11, 12q24 and 15q22 that are associated with risk of CAD or its major complication, myocardial infarction [3–9]. To date, few GWAS have been conducted in Chinese populations. Thus, the susceptibility genes that significantly contribute to the development of CAD in the majority of Chinese Han population remain poorly characterized.

The purpose of the present study was to find out novel susceptibility genes for CAD in Chinese Han population. Our design included a discovery study (stage 1), a validation study (stage 2) and a replication study (stage 3). In stage 1, we performed GWAS in “extreme individuals” from Shandong cohort and a pathway-based candidate gene case-control association study in a Shanghai cohort in combination with the data from a British population by the Wellcome Trust Case Control Consortium (WTCCC). In stage 2, we selected 13 SNPs for validation and identified a novel susceptibility locus *rs700926* in natriuretic peptide receptor C (*NPR-C*). In stage 3, we performed case-control association studies of tag SNPs to further validate new susceptibility loci of *NPR-C* for CAD in 6 larger cohorts from northern, southern and central China.

## RESULTS

### Study population characteristics

The demographical and clinical characteristics of the discovery, validation and replication populations are summarized in Table 1. The prevalence of established cardiovascular risk factors (except for gender) was significantly higher in controls than that in cases. In candidate gene association study, a lower prevalence of hypertension (62.1% *versus* 67.2%), a higher prevalence of diabetes (27.3% *versus* 6.8%) and hyperlipidemia (67.2% *versus* 62.2%), and a higher percentage of ever and /or current smoking (39.6% *versus* 26.3%) were observed in cases than in controls. In the validation and replication study populations, as anticipated, the prevalence of established cardiovascular risk factors was significantly higher in cases than that in controls. Overall, the patient characteristics in the present study were typical for those undergoing coronary angiography for the evaluation of CAD, with a male preponderance (over 61.4%) and a high prevalence of diabetes (over 17.6%), hypertension (over 46.7%), hyperlipidemia (over 8.9%) as well as ever and /or current smoking (over 15.4%). Moreover, as expected, the serum TC levels were significantly higher but the serum HDL-C levels lower in cases than in controls.

**Table 1:** Characteristics of the study population

Population		Age^*^	Gender Female *n*(%)	Hypertension *n*(%)	Diabetes *n*(%)	BMI kg/m^2^	Hyperlipidemia *n*(%)	Ever/current Smokers *n*(%)	MI *n*(%)	PCI *n*(%)	CABG n(%)
**Dis-Shandong**	Case(*n* = 100)	44.9±4.81	20(20.0)	20(20.0)	4(4.0)	25.1±8.5	4(4.0)	32(32.0)	42(42.0)	56(56.0)	4(4.0)
	Control(*n* = 100)	62.3 ±6.7	22(22.0)	78(78.0)	26(26.0)	NA	32(32.0)	58(58.0)	0(0)	0(0)	0(0)
	*p*	*<0.001*	*0.27*	*<0.001*	*<0.001*	*<0.001*	*<0.001*	*<0.001*	*<0.001*	*<0.001*	*<0.001*
**Dis- Shanghai**	Case(*n* = 293)	63.3±10.5	113(38.6)	182(62.1)	80(27.3)	24.1±2.8	197(67.2)	116(39.6)	NA	NA	NA
	Control(*n* = 293)	63.3±10.6	113(38.6)	197(67.2)	20(6.8)	23.9±3.2	182(62.2)	77(26.3)	0(0)	0(0)	0(0)
	*p*	*0.541*	*0.793*	*<0.001*	*<0.001*	*0.282*	*<0.001*	*<0.001*	*NA*	*NA*	*NA*
**Val-Shandong**	Case(*n* = 480)	59.9±9.5	116(24.2)	276(57.5)	104(21.7)	26.0±3.2	132(27.5)	258(53.8)	301(62.7)	257(53.5)	22(4.6)
	Control(*n* = 469)	40.8±12.8	194(41.4)	3(0.006)	2(0.004)	24.8±3.5	1(0.002)	119(25.3)	0(0)	0(0)	0(0)
	*p*	*<0.001*	*<0.001*	*<0.001*	*<0.001*	*<0.001*	*<0.001*	*<0.001*	*<0.001*	*<0.001*	*0.000*
**Val- Hubei**	Case(*n* = 694)	59.8±11.8	156(22.5)	425(61.2)	134(19.3)	24.4±3.5	79(11.4)	389(56.1)	319(46.0)	247(35.6)	9(1.3)
	Control(*n* = 979)	49.5±10.2	598(59.0)	4(0.004)	3(0.003)	22.8±2.6	1(0.001)	19(1.9)	0(0)	0(0)	0(0)
	*p*	*<0.001*	*<0.001*	*<0.001*	*<0.001*	*<0.001*	*<0.001*	*<0.001*	*<0.001*	*<0.001*	*<0.001*
**Rep- Shanxi**	Case(*n* = 392)	60.1±12.3	83(21.2)	183(46.7)	69(17.6)	24.7±4.2	16(17.4)	197(50.3)	101(25.8)	276(70.4)	7 (1.8)
	Control(*n* = 236)	40.4±18.1	115(48.7)	1(0.004)	1(0.004)	22.74±3.4	1(0.004)	35 (14.8)	0(0)	0(0)	0(0)
	*p*	*<0.001*	*<0.001*	*<0.001*	*<0.001*	*<0.001*	*<0.001*	*<0.001*	*<0.001*	*<0.001*	*<0.001*
**Rep- Sichuan**	Case(*n* = 480)	65.9±10.8	90(18.8)	253(52.7)	100(20.8)	24.6±5.8	67(14.0)	273(56.9)	140(29.2)	464(96.7)	8(1. 7)
	Control(*n* = 460)	54.1±16.1	245(53.2)	2(0.004)	3(0.006)	23.7±4.7	1(0.002)	55 (12.0)	0(0)	0(0)	0(0)
	*p*	*<0.001*	*<0.001*	*<0.001*	*<0.001*	*<0.001*	*<0.001*	*<0.001*	*<0.001*	*<0.001*	*<0.001*
	Case(*n* = 1024)	65.3±10.8	272(26.6)	662(64.7)	182(17.8)	24.1±2.8	91(8.9)	342(33.4)	NA	NA	NA
**Rep-Center2**	Control(*n* = 711)	60.2±9.0	386(54.3)	348(49.0)	58(8.2)	24.1±4.1	29(4.1)	151(21.2)	0(0)	0(0)	0(0)
	*p*	*<0.001*	*<0.001*	*<0.001*	*<0.001*	*<0.001*	*<0.001*	*<0.001*	NA	NA	NA

Age of cases and Age of controls, age at the time of enrollment. Data for age and BMI are shown as mean ± standard deviation. We used χ^2^ tests to compare the differences between gender, hypertension, type 2 diabetes and smoking status. We used a Student's *t* test to compare the means of the cases and controls. NA, no data; NS: no significant; BMI, body mass index;

* Age in years.

### SNPs potentially associated with CAD

In the discovery stage, 100 “extreme cases” and 100 “extreme controls” recruited from Shandong were genotyped using Infinium HumanOmnizhonghua-8 BeadChip (Illumina). We constructed quantile-quantile (Q-Q) plot for this population using the genome-wide genotyping data (Supplementary Figure S.1A). To ensure that case and control groups were genetically matched, in addition to close examination of their geographic origins, multidimensional scaling (MDS) was used to exclude population outliers (Supplementary Figure S.1B), the result of which was further confirmed by principal-component analysis (PCA), which showed minimal evidence for population stratification. A total of 8,7032 SNPs failed to achieve a call rate of 90% and thus were excluded from further analysis, whereas 78,5229 SNPs in 100 “extreme cases” and 100 “extreme controls” passed quality control and entered final statistical analysis using the Cochran-Armitage trend test to examine the genotype-phenotype association under an additive model. After genomic control (GC) with an inflation factor of 1.08 [6], the association results did not change significantly. Supplementary Figure S.2 provided a plot of the meta-analysis *p* values by chromosome position. Given a small sample size of 200 and the design of “extreme cases and controls”, a *P* value 1'10^−4^ was considered strong evidence for association in this discovery stage, although the a priori threshold for genome-wide significance was 5'10^−8^. Nonetheless, the relative small sample size in our GWAS study may result in a negative result due to limited statistical power. To avoid possible bias caused by this arbitrary selection, for SNPs with weaker associations (*P* value between 10^−4^ and 10^−3^, Supplementary Figure S.2), we also searched signal pathway-based candidate genes related to atherosclerosis and CAD reported from previous publications.^2^ Combined with the reported candidate gene and the earlier reports of the highthroughput dataset from a British population by WTCCC (http://www.wtccc.org.uk/) in relation to CAD, we screened a total of 120 SNPs representing 49 genomic regions of human chromosome 1, 2, 3, 4, 5, 6, 7, 8, 9, 10, 11, 12, 13, 16, 18, 19, 20 and 22 (Supplementary Table S.1*)*. These SNPs were genotyped and analyzed in 596 unrelated Han individuals (293 CAD patients and 293 controls) in Dis-Shanghai cohort. In addition to the known SNPs, we focused on the 25 SNPs representing 7 genomic regions (*HFE:* rs2071303, rs17596719, rs6918586*; CAT:* rs769214, rs1049982, rs11032699, rs11032700, rs2179625, rs7104301*; HOMX1:* rs2071748, rs4820192, rs5755718, rs5755720*; CXCL9:* rs2276886; *PON3*: rs978903*; LAMA4:* rs1016825, rs736160, rs6568719, rs2237248, rs9320402, rs4571602*; NPR-C:* rs12697273, rs10066436, rs3828586, rs10061804) using a significant threshold (Supplementary Table S.1). These regions and SNPs were further analyzed from the GWAS scan data with any of the genotype, allele, dominant, and recessive models for subsequent cross-platform validation using Sequenom. The results demonstrated that only 12 SNPs in 3 regions (rs10074149, rs11749133, rs16890187, rs1833530, rs696835 and rs973079 in NPR-C, rs2282854, kgp9060646 and kgp2840023 in LAMA4, rs2074352, rs17884000 and rs17774169 in PON3) showed weak association at stage 1 with the genotype, allele, dominant, or recessive models (Supplementary Table S.2). Furthermore, these *P*-values were below or near the genome-wide significance level of *5'10^−4^*. In contrast, other 56 SNPs in HMOX1, CXCL9, HFE and CAT genes showed no significant association with CAD by GWAS in Dis-Shandong cohort (Supplementary Table S.2). This discrepancy may result from the limited sample size of our GWAS, although its population was highly selected from “extreme individuals”.

To further test their potential association with CAD in a larger population, 13 tag SNPs (rs2071303 and rs2794719 in *HFE;* rs554576, rs524154 and rs7947841 in *CAT;* rs2071749 in *HOMX1;* rs2276886 and rs2869460 in *CXCL9;* rs2057682, rs7787187 and rs11977702 in *PON3;* rs6568719 in *LAMA4;* rs700926 in *NPR-C*) that have relatively strong representativeness for these 7 genomic regions, respectively, were selected from the HapMap database and considered for genotyping analysis in the two independent validation populations from Northern (Val-Shandong Cohort) and Southern (Val-Hubei Cohort) China. Except for rs700926 in *NPR-C*, none of the 12 SNPs being validated (*HFE:* rs2071303, rs2794719*; CAT:* rs554576, rs524154, rs7947841*; HOMX1:* rs2071749*; CXCL9:* rs2276886, rs2869460*; PON3:* rs2057682, rs7787187, rs11977702) showed significant association with CAD in Val-Shandong and Val-Hubei cohorts (Supplementary Table S.3), and thus these 12 SNPs did not undergo further analysis.

### Novel susceptibility locus for CAD

Importantly, rs700926 in *NPR-C* was identified to be consistently associated with CAD in 2 cohorts from Shandong and Hubei populations (Table 2). We further analyzed the association between rs700926 and CAD in other 2 cohorts from Northern (Rep-Shaanxi) and Southern (Rep-Sichuan) China and identified a consistent association with CAD, even in combined population from 4 geographically different regions with *P* = 5.0×10^−07^ and OR = 1.36 for allele G (Table 2). To further investigate how the SNP alleles are interacted with traditional risk factors (age, gender, smoking, hypertension and diabetes) in conferring genetic risks for CAD, we conducted genotypic association analysis using multivariate logistic regression analysis. A significant association still existed after adjustment for conventional risk factors for CAD (*P*_adj_ = 1.2×10^−6^) (Table 3). With our sample size and with the alpha level set at 0.05, there was at least 80% power for detection of SNPs with OR ≥ 1.3. These results suggest rs700926 in *NPR-C* located in Chr 5p confers a highly significant risk of CAD in the northern and southern Chinese Han populations and that rs700926 may represent a new susceptibility locus for CAD.

**Table 2:** Allelic association of SNPs of *NPR-C* with coronary artery disease in Rep-Shandong, Rep-Hubei, Re-Sichuan and Re-Shaanxi cohorts

SNP_ID	Minor Allele	Re-Shandong				Re-Hubei				Re-Sichuani				Re-Shaanxi			
		MAF(%)*		OR(95%CI) †	P-obs ‡	MAF(%)*		OR(95%CI) †	P-obs ‡	MAF(%)*		OR(95%CI) †	P-obs ‡	MAF(%)*		OR(95%CI) †	P-obs ‡
		Case	Control			Case	Control			Case	Control			Case	Control		
**rs700926**	G	23.6	19.4	1.34(1.07-1.52)	3.2E-4	26.0	19.1	1.42(1.17-1.59)	5.4E-5	24.1	19.6	1.40(1.08-1.51)	1.4E-5	26.3	21.4	1.25(0.95-1.44)	2.2E-4
**rs1833529**	G	22.2	19.3	1.30(1.15-1.48)	1.5E-4	23.4	17.9	1.35(1.09-1.57)	1.5E-5	21.0	15.0	1.45(1.05-163)	2.9E-5	23.7	17.5	1.38(1.01-1.45)	8.5E-4
**rs2270915**	G	20.1	14.9	1.43(1.12-1.83)	3.8E-3	20.9	17.6	1.23(1.10-152)	0.05	21.0	15.0	1.51(1.10-1.73)	1.7E-3-	21.5	16.8	1.38(1.10-1.78)	5.1E-4
**rs17541471**	C	17.2	14.0	1.29(1.05-1.61)	0.02	17.5	13.4	1.31(1.08-1.69)	0.01	17.0	13.0	1.30(1.03-1.61)	0.07	18.0	13.2	1.40(1.05-1.65)	0.01
**rs3792758**	T	17.1	13.1	1.32(1.10-1.61)	3.0E-3	18.4	12.8	1.54(1.20-1.99)	7.7E-4	17.0	12.5	1.61(1.10-1.82)	5.1E-3	17.5	13.0	1.43(1.10-1.75)	1.0E-3
**rs696831**	T	19.1	15.4	1.38(1.12-1.75)	0.02	17.3	13.7	1.32(1.02-1.70)	0.03	18.3	15.0	1.40(1.06-1.70)	0.01	18.1	13.4	1.37(1.12-1.75)	0.04
**rs7715279**	A	12.1	11.2	1.20(0.93-1.30)	0.49	11. 7	9.6	1.25(0.94-1.65)	0.12	11.2	10.0	1.23(0.95-1.37)	0.13	12.8	12.6	0.98(0.69-1.40)	0.93
**rs6450922**	G	25.4	24.5	1.25(1.02-1.25)	0.65	26.1	25.5	1.29(1.00-1.67)	0.05	25.3	24.9	1.20(1.00-1.20)	0.98	251	24.6	1.18(0.95-1.20)	0.84
**rs10941022**	T	13.6	14.5	0.92(0.71-1.21)	0.57	12.7	11.6	1.11(0.90-1.37)	0.34	14.6	13.9	1.10(0.98-1.27)	0.81	14.1	13.6	1.01(0.98-1.28)	0.71
**rs976576**	G	29.2	27.0	1.00(0.85-1.14)	0.21	28.5	27.0	1.05(0.95-1.15)	0.54	28.0	27.5	1.02(0.79-1.10)	0.69	29.3	27.1	1.12(0.98-1.21)	0.32

* MAF: minor allele frequency.

† OR, odds ratio. CI, confidence interval.

‡ *P*-obs, uncorrected *P* value.

**Table 3:** Allelic association of SNPs of *NPR-C* with coronary artery disease in 4 combined cohorts (Rep-Shandong, Rep-Hubei, Re-Sichuan and Re-Shaanxi)

SNP_ID	Position	Allele	MAF(%)		*p*-HW *	OR(95%CI) †	*P*-obs ‡	*P*-adj ^x^
			Case	Control				
**rs700926**	32745283	G	24.9	19.7	0.37	1.36(1.17-1.57)	5.0E-7	1.2E-6
**rs1833529**	32734140	G	22.9	18.5	0.45	1.31(1.12-1.52)	3.0E-6	1.0E-5
**rs2270915**	32786389	G	20.5	16.3	0.89	1.33(1.13-1.55)	5.7E-6	9.0E-6
**rs17541471**	32755589	C	18.4	12.8	0.33	1.54(1.20-1.99)	8.2E-6	2.0E-5
**rs3792758**	32745752	T	22.5	19.0	0.53	1.24(1.04-1.47)	0.014	0.026
**rs696831**	32725531	T	17.1	13.7	0.67	1.30(1.03-1.64)	0.029	0.041
**rs7715279**	32689875	A	11.8	11.0	0.78	1.08(0.89-1.31)	0.454	0.598
**rs6450922**	32689718	G	25.3	24.8	0.82	1.29(1.00-1.67)	0.053	0.071
**rs10941022**	32691416	T	12.7	11.6	0.98	1.11(0.90-1.37)	0.343	0.542
**rs976576**	32722499	G	36.9	37.1	0.74	0.99(0.86-1.14)	0.752	0.915

* *P*-HW, *P* value for Hardy-Weinberg disequilibrium analysis.

† OR, odds ratio, CI, confidence interval.

‡ *P*-obs, uncorrected *P* value.

x *P*-adj, *P* value obtained after adjustment for gender, age, hypertension and diabetes.

### Association analysis between common variants of NPR-C and CAD

To assess whether common variants in the *NPR-C* gene were associated with sporadic CAD in the Chinese population, 9 tag SNPs of *NPR-C (rs1833529, rs2270915, rs17541471, rs3792758, rs696831, rs7715279, rs6450922, rs10941022* and *rs976576)* were firstly genotyped by the Shandong research group in 4 cohorts from Northern (Rep-Shandong and Rep-Shaanxi) and Southern (Rep-Hubei and Rep-Sichuan) China, respectively (Supplementary Figure S.3). Among these SNPs, 5 tag SNPs (*rs1833529, rs2270915, rs17541471, rs3792758 and rs696831*) exhibited a significant association with CAD in Rep-Shandong cohort, whereas additional 4 SNPs (*rs7715279, rs6450922, rs10941022 and rs976576*) showed no significant association with CAD. Then the above 5 positive SNPs were further tested in Rep-Hubei, Rep-Sichuan and Rep-Shaanxi cohorts, respectively, and the results showed positive association with CAD (Table 2). Thereafter, we combined the 4 cohorts together and performed further association analysis, which again demonstrated positive association with CAD (Table 3). rs3792758 with larger OR ratios (1.45(1.10-1.75)) and lower *p*-values (*p* = 1.0^−3^) were observed in Rep-Shaanxi cohorts (Table 2), a result induced probably by the small sample size of this cohort. In addition, the Shanghai research group genotyped rs2270915 and additional 10 tag SNPs (*rs9716700, rs11750438, rs6889608, rs10036648, rs12697273, rs10066436, rs3828586, rs2062708, rs10061804 and rs7730564*) in Rep-Center 1 and 2 populations (Supplementary Figure S.4). Of them, only rs12697273 and rs10066436 of *NPR-C* showed a consistent association with CAD in these two cohorts (Table 4), and these associations remained significant even after adjustment for the statistically significant covariates of age, gender, hypertension, diabetes, smoking, total cholesterol, triglyceride, high-density lipoprotein cholesterol (HDL-C) and low-density lipoprotein cholesterol (LDL-C). Four SNPs of them (rs2270915, rs10036648, rs10061804 and rs3828586) exhibited a positive association with CAD only in Rep-Center 1 but not in Rep-Center 2 populations. Furthermore, there is no mutation of rs9716700 in the promoter region of the *NPR-C* gene in the central China population. In each of the 4 cohorts, the remaining 4 SNPs in introns (rs11750438, rs6889608, rs7730564 and rs2062708) showed no significant association with CAD. Though rs700926 has been identified to be associated with CAD in the northern and southern China population, rs10061804 and rs3828586, which are well representative of rs700926 (both *r*^2^ = 1.0) (Figure 2), did not show similar association in the two central China cohorts. According to Hardy-Weinberg equilibrium analysis, rs700926 is also well representative of rs1833529, rs17541471 and rs3792758 (Figure 2). Thereafter, the two new susceptibility loci (rs12697273 and rs10066436) identified in the central China population were further replicated in Rep-Shandong and Rep-Hubei cohorts, and the result showed no significant association between these two SNPs and CAD (Table 5). In addition, we performed association analysis between the remaining SNPs not associated with CAD and the known risk factors for CAD and did not find any significant association between them (Table 3).

**Table 4:** Allelic association of SNPs of NPR-C with coronary artery disease in Central Chinese Han population

Population	SNP_ID	Position	MAF(%)*		Allele	OR(95%CI) †	*P*-obs ‡	*P*-adj ^x^
			case	control				
**Cohort 1**	rs9716700	32711633	0	0	A		1	
	rs11750438	32716535	0.143357	0.121993	T	0.83(0.59-1.17)	0.297177	
	rs6889608	32719693	0.305263	0.261168	T	0.80(0.62-1.04)	0.0734387	
	rs10036648	32724078	0.166084	0.146048	G	1.16(0.85-1.60)	0.040828	
	rs12697273/10037355	32730464	0.258741	0.185567	T	0.65(0.49-0.86)	0.0088942	
	rs10066436	32740346	0.255245	0.183849	T	0.66(0.50-0.87)	0.0111536	
	rs3828586	32746547	0.265734	0.197595	G	1.47(1.12-1.94)	0.021309	
	rs10061804	32748637	0.265734	0.197595	A	0.68(0.52-0.90)	0.021309	
	rs2062708	32753672	0.185315	0.146048	C	1.33(0.97-1.82)	0.142288	
	rs7730564	32767727	0.185315	0.14433	G	1.35(0.99-1.84)	0.118293	
	rs2270915	32786389	0.20979	0.185567	G	1.17(0.87-1.56)	0.0460445	
**Cohort 2**	rs9716700	32711633	0	0.000661	A		0.424719	
	rs10036648	32724078	0.171329	0.155378	G	1.12(0.94-1.35)	0.290962	
	rs12697273/10037355	32730464	0.1865	0.220961	T	1.24(1.05-1.46)	0.0107192	
	rs10066436	32740346	0.182548	0.22237	T	1.28(1.08-1.51)	0.00484991	
	rs3828586	32746547	0.202663	0.236037	G	0.82(0.70-0.97)	0.0592019	
	rs10061804	32748637	0.203156	0.225033	A	1.14(0.97-1.34)	0.295754	
	rs2270915	32786389	0.174729	0.181941	G	0.95(0.80-1.13)	0.688235	
**Combined**	rs12697273/10037355	32730464						1.2E-3
	rs10066436	32740346						1.5E-4

* MAF: minor allele frequency.

† OR, odds ratio. CI, confidence interval.

‡ *P*-obs, uncorrected *P* value.

x *P*-adj, P value obtained after adjustment for gender, age, hypertension, and diabetes.

**Table 5:** Allelic association of *rs12697273* and *rs10066436* with coronary artery disease in Rep-Shandong and Rep-Hubei cohorts

SNP_ID	Population	MAF(%)*		Allele	OR(95%CI) †	*P*-obs ‡
		case	control			
rs12697273	Rep-Shandong	0.17314	0.196203	T	0.86(0.64-1.15)	0.3111427
	Rep-Wuhan	0.209239	0.188716	T	1.14(0.90-1.14)	0.28547
	Combined	0.18559	0.190476	T	0.94(0.80-1.11)	0.48005526
rs10066436	Rep-Shandong	0.19237	0.201721	T	1.07(0.80-1.41)	0.375217
	Rep-Wuhan	0.213043	0.192308	T	1.14(0.85-1.52)	0.387946891
	Combined	0.201325	0.193023	T	1.10(0.83-1.40)	0.573591

* MAF: minor allele frequency.

† OR, odds ratio. CI, confidence interval.

‡ *P*-obs, uncorrected *P* value.

**Figure 1:**
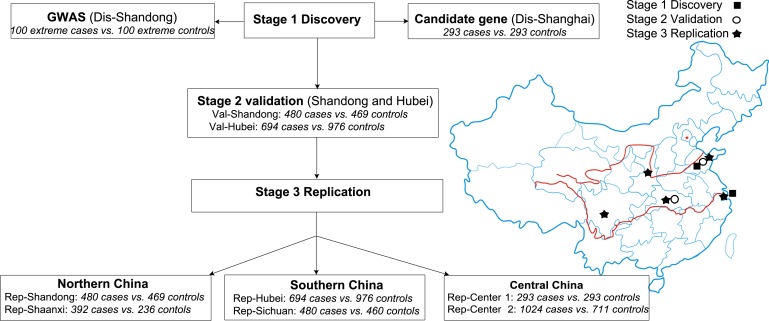
Flow chart of the present study The stage 1 discovery study enrolled two cohorts of populations: a GWAS-based population in Shandong including 200 “extreme individuals” (Dis-Shandong) and a candidate gene-based study population including 596 individuals in Shanghai (Dis-Shanghai). The stage 2 validation study consisted of 480 cases and 469 controls from Shandong (Val-Shandong) and 694 cases and 979 controls from Hubie (Val-Hubei). Stage 3 replication studies of rs700926 and the nine tag SNPs of NPR-C included 5 independent cohorts: Rep-Shanghai, Rep-Shandong, Rep-Shaanxi, Rep-Hubei and Rep-Sichuan in China. The stage 3 replication study included 480 cases and 469 controls in Shandong (Rep-Shandong), 392 cases and 236 controls in Shaanxi (Rep-Shaanxi), 694 cases and 976 controls in Hubei (Rep-Hubei), 480 cases and 460 controls in Sichuan (Rep-Sichuan), 293 cases and 293 controls in Cohort 1 in Central China (Rep-Center 1) and 1024 cases and 711 controls in Cohort 2 of Cnetral China (Rep-Center 2). Rep-Shandong is the same as Val-Shandong, Rep-Hubei is the same as Val-Hubei, and Rep-Center 1 is the same as Dis-Shanghai.

**Figure 2:**
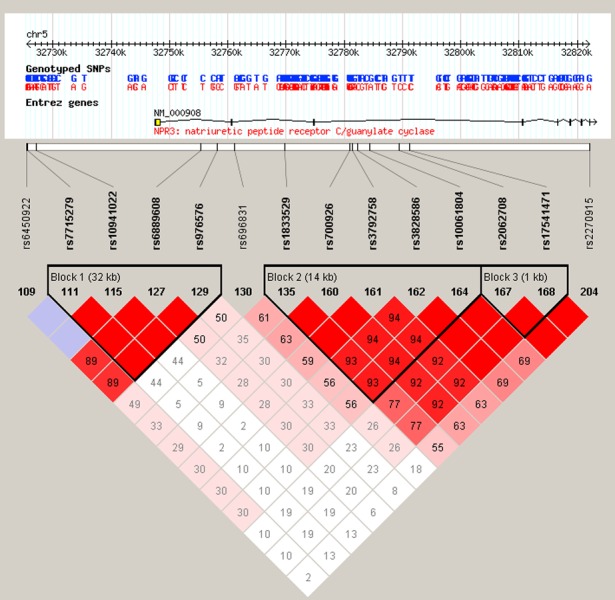
Block structure of linkage disequilibrium for the tag SNPs in the natriuretic peptide receptor C gene region (NPR-C) on chromosome 5p13.3 The linkage disequilibrium (LD) of the tag SNPs with other SNPs were assessed respectively using the data from HapMap by a D' > 0.9 and an r^2^ > 0.7 as standard. Note that rs700926 has relatively strong representativeness of rs1833529, rs17541471, rs3792758 and rs3828586.

### Association of the risk allele G of rs700926 with expression of NPR-C mRNA in human peripheral blood leukocytes

**Figure 3:**
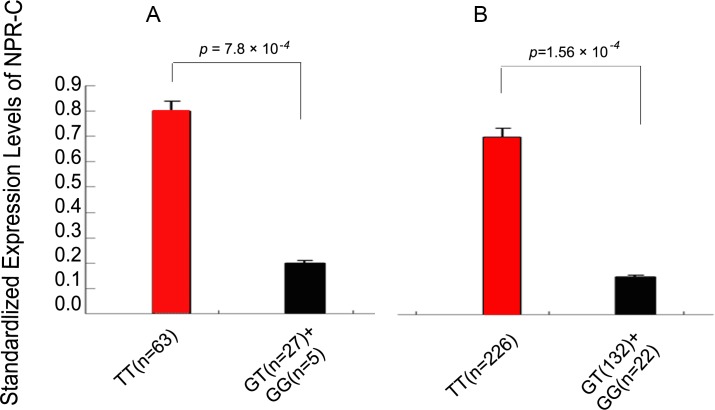
Association of the risk allele G of rs700926 with decreased expression of NPR-C mRNA assuming a dominant model Because rs700926 is located near intron 1 of NPR-C, we hypothesized that it may affect the expression levels of the NPR-C mRNA. **A.** 5 individuals with a minor genotype of GG, 27 individuals with genotype GT and 63 individuals with genotype TT of rs700926 were selected for RT-PCR analysis. **B.** To validate the finding of a quantitative expression trait locus from the initial analysis, a replication study was also performed in an independent population of 380 randomly selected individuals from the replication populations (northern and southern Chinese).

Because these positively associated SNPs (*rs700926, rs1833529, rs2270915, rs17541471, rs3792758, rs696831, rs12697273* and *rs10066436*) are not located in the promoter or extron of NPR-C, they may not influence directly the expression levels of NPR-C mRNA. Compared with other seven SNPs, rs700926 is located near intron 1 of NPR-C, and thus we hypothesized that it may affect the expression levels of the NPR-C mRNA. To test this hypothesis, we used real-time PCR analysis to measure the NPR-C expression levels in human peripheral blood leukocytes from 95 individuals with different genotypes. As shown in Figure 3A, the mRNA expression levels of NPR-C were significantly higher in 63 individuals with the TT genotype than in 32 individuals with the GG or GT genotype. To further confirm these results, we performed a replication study with a randomly selected group of 380 individuals. As shown in Figure 3B, the mRNA expression level of NPR-C was also significantly higher in 226 individuals with the TT genotype than in 254 individuals with the GG or GT genotype.

## DISCUSSION

In the present study, we performed a multicenter GWAS, pathway-based candidate gene association and replication studies in 6 geographically different Chinese Han populations and identified new susceptibility loci of *NPR-C* that was specifically associated with CAD.

In the first stage of the study, we found sufficient evidence for a potentially novel SNP rs700926 in NPR-C gene associated with CAD in Han Chinese. Then we identified other 5 tag SNPs *(rs1833529, rs2270915, rs17541471, rs3792758 and rs696831)* associated with CAD simultaneously in the northern and southern Han Chinese population. In addition, another 2 novel tag SNPs *(rs12697273 and rs10066436)* in *NPR-C* gene also showed significant association with CAD in the central Han Chinese. Noticeably, these significant associations remained significant even after adjustment for conventional risk factors of atherosclerosis (age, gender, smoking, hypertension, diabetes and hyperlipidemia), suggesting that the contribution of these polymorphisms to the risk of CAD is independent of conventional risk factors. In addition to the strict criteria used for defining CAD and normal phenotypes in this study, all CAD patients and normal controls were verified to be genetically unrelated by examining their familial relationships. In order to further validate these findings, we recruited a large number of subjects from northern, southern and central China, who were well representative of the general Han Chinese population [10]. Importantly, cross-replication of the results from 5 independent populations may decrease the rate of false positives. Therefore, our results are unlikely due to a specific cohort effect and are able to identify significant SNPs associated with CAD despite the small sample size in the discovery study.

As the largest ethnic group in mainland China, Han Chinese is a geographically and genetically heterogeneous population and has subpopulation structures, which may have considerable effect on the design and interpretation of replication studies [10]. In the present study, we identified 6 associated SNPs of NPR-C (*rs700926, rs1833529, rs2270915, rs17541471, rs3792758* and *rs696831)* in the northern and southern Chinese Han population and 2 different associated SNPs *(rs12697273* and *rs10066436)* of *NPR-C* in the central Chinese Han population. As *rs3828586*, *rs10061804* and *rs700926* have strong LD association with each other, *rs700926* is well representative of *rs3828586* and *rs10061804*. Although *rs700926* and *rs2270915* have been verified to be associated with the risk of CAD in the northern and southern Han Chinese populations, *rs3828586*, *rs10061804* and *rs2270915* didn't show a consistent association with CAD in the central Han Chinese population. Similarly, another two CAD-associated SNPs *(rs12697273 and rs10066436)* found in the central Chinese Han population did not show a consistent association with CAD in the northern and southern Han Chinese. These results further demonstrated the diversity of the Chinese human genome. Furthermore, allele effects in the central Han Chinese were consistently weaker than those in the northern and southern Han Chinese, suggesting that there is a greater genetic difference between central and northern or southern Chinese populations. In the prior GWAS for CAD in Chinese population, genome-wide significant associations of loci in or near C6orf105, TTC32-WDR35, GUCY1A3, C6orf10-BTNL2 and ATP2B1 with CAD were reported by Wang F et al and Lu XF et al independently [11, 12]. However, the new loci identified by Wang F et al were not confirmed by Lu XF et al. Similarly, these new loci were not verified in our cohorts. These inconsistent results may be due to different genetic backgrounds in geographically different Chinese Han population. In the study of Wang F et al, the subjects were recruited from the northern and central China, whereas in the study of Lu XF et al, all participants in the discovery stage and the majority of cases and controls in the replication stage were enrolled from the northern China. By comparison, our subjects were recruited from the northern, southern and central Han Chinese who is well representative of the general Han Chinese population. In addition, normal controls were defined only by normal history and physical examination in previous studies, whereas in our study, only those with normal history, physical examination, blood chemistry and angiography were included as normal controls. Thus, the strict criteria for defining CAD patients and normal controls may explain in part the different results between our and previous studies.

The *NPR-C* gene resides on human chromosome 5p14-p13, and *NPR-C* represents the most widely and abundantly expressed natriuretic peptide receptor (NPR), with a tissue distribution that includes most of the major endocrine glands, lungs, kidneys and vessels [13]. In particular, *NPR-C* is the most common type of NPR in endothelial cells [14]. Although *NPR-C* was traditionally regarded as a clearance receptor of natriuretic peptides (NPs), recent studies have revealed multiple effects of *NPR-C* on different cells and organs [14, 15] and most of these effects were enhanced by C-type natriuretic peptide (CNP) stimulation rather than by A-type atrial natriuretic peptide (ANP) and B-type natriuretic peptide (BNP) [13]. Recently, a study in 200,000 European descents also showed an association between rs1173771 in NPR3-C5orf23 with hypertension [16]. However, the association between *NPR-C* gene and CAD has never been reported in any population studies. In the current study, 6 SNPs of NPR-C (*rs700926, rs1833529, rs2270915, rs17541471, rs3792758* and *rs696831*) were identified to be associated with CAD in the northern and southern Chinese populations and another two SNPs *(rs12697273* and *rs10066436*) found to be associated with CAD in the central Chinese population. Moreover, multivariate logistical regression analysis showed that the association between these SNPs and CAD remained significant even after adjustment for conventional risk factors of CAD including hypertension.

The molecular mechanisms underlying the association between NPR-C gene polymorphism and CAD are unclear but preliminary results revealed a potential role of *NPR-C* in the pathogenesis of atherosclerosis. A recent study showed that *NPR-C* expression was increased in neointimal smooth muscle cells in patients receiving percutaneous coronary intervention [17]. Molecular imaging technique demonstrated the presence of NPR-C near the luminal surface of atherosclerotic plaques and in VSMCs [18]. Moreover, *NPR-C*-dependent ERK 1/2 phosphorylation activated the vasoprotective effect of CNP, resulting in augmentation of endothelial cell proliferation and inhibition of VSMCs growth [19]. Furthermore, Fox AA et al found that 4 *NPR-C* SNPs (*rs700923, rs16890196, rs765199, rs700926*) were associated with left ventricular dysfunction after coronary artery bypass grafting and able to predict patient outcome when combined with NPRC SNPs [20]. In this study, we also detected NPR-C expression in leukocytes from individuals with different genotypes. Although *rs700926* near intron 1 of NPR-C may not directly influence the expression levels of the NPR-C mRNA, we found that the mRNA expression level of NPR-C in human peripheral blood leukocytes was significantly higher in individuals with the TT genotype than those with the GG or GT genotype. These findings are consistent with established effects of NPR-C variant on CAD.

We also investigated the association of some conflicting SNPs (*HFE: rs2071303, rs2794719; CAT: rs554576, rs524154, rs7947841; HOMX1: rs2071749; CXCL9: rs2276886, rs2869460; PON3: rs2057682, rs7787187, rs11977702; LAMA4: rs6568719*) with CAD in our Chinese populations. Recently, a case-control study from Hubei province of China found that SNP *rs9366637* in HFE gene was associated with higher CAD risk in their study populations [21]. Although our study found a significant association between SNP *rs2794719* in *HFE* gene and CAD from Hubei cohort, this association was not confirmed in the southern (Rep-Sichuan) and northern (Rep-Shandong and Rep-Shaanxi) Han Chinese populations. Furthermore, SNP *rs2071303* in HFE gene showed no significant association with CAD in these cohorts. Previous studies investigated the role of the polymorphisms of *Heme oxygenase-1 (HMOX1)*, the only inducible form of heme-oxygenases, in the development of CAD. However, the results of these studies were conflicting [22, 23]. Similar to the result of Lin R et al [24], our study in the southern and northern Han Chinese populations showed insignificant association between SNP *rs2071749* in *HMOX-1* gene and CAD. Experimental studies also showed that paraoxonases (PONs) including PON1, PON2 and PON3 may prevent low density lipoprotein cholesterol (LDL-C) from peroxidation and play a protective role in atherogenesis [25]. Rull A et al [26]. reported a positive association between serum PON3 and β-2-microglobulin, CCL2 and high-sensitivity C-reactive protein in CAD patients but insignificant differences in PON3 gene promoter polymorphisms and their haplotypes between CAD patients and controls, indicating that these associations were not genetically determined. We also genotyped 3 tag SNPs (*rs2057682, rs7787187* and *rs11977702*) in *PON3* gene and identified no association between these SNPs and CAD.

There are several limitations of our study that must be acknowledged. First, the sample size in the discovery stage was small. However, the sample size in our validation and replication stages was large enough to rule out the possibility that our findings of *NPR-C* as a novel susceptibility locus of CAD in the Chinese Han population were false positive. Second, some positive SNPs identified in some cohorts of the current study became negative in other cohorts. A possible explanation is that different populations may have different modifier genes, lifestyles, or gene-gene and gene-environment interactions. Third, although the association between *NPR-C* polymorphism and CAD was highly significant in our study population, other variants in nearby genes in strict linkage disequilibrium with *NPR-C* polymorphism may be responsible for the observed genetic association. It is likely that the positive SNPs identified in this study may be a simple marker for CAD whereas the true causative SNP in LD with these positive SNPs at 5p13 may be located at a considerably large distance from these SNPs. Fourth, because it was difficult to obtain sufficient human coronary artery specimens, the association of the risk allele of *rs700926* with expression of *NPR-C* mRNA in human coronary artery was not investigated in this study. Finally, our findings are limited to Chinese Han population and further studies are required to confirm the role of *NPR-C* as a novel susceptibility locus of CAD in other Asian or Western populations.

In conclusion, our study showed 8 SNPs including *rs700926, rs1833529, rs2270915, rs17541471, rs3792758, rs696831, rs12697273* and *rs10066436* in *NPR-C* gene on chromosome 5p13 are associated with increased susceptibility to CAD in Chinese Han population. This finding may lead to the discovery of novel pathways in atherogenesis and hence, new preventive and therapeutic targets in CAD patients. Further studies are warranted to identify the true causative variant(s) for CAD at this new 5p13 CAD locus.

## MATERIALS AND METHODS

### Power analysis

Before implementation of this study, we performed a statistical power analysis using the PS version 3.0.43 to ascertain whether the recruited samples could provide adequate power for identifying the association between the modest-effect-size SNPs and CAD, provided that the NPR-C SNPs locus confers the same size of risk for development of CAD in Chinese Han population. Under the population parameter settings of the effect size of odd ratios of 1.30 and the allelic frequency of 0.16 chiefly derived from Hapmap database, and on the basis of our preliminary experiment, our samples with 3363 well-characterized CAD cases and 3148 healthy controls can provide a statistical power of 98.2% and 92.9% at the nominal Type I error rate of 0.05 and 0.01, respectively. The power analysis indicates that our Han Chinese sample size is sufficient for identifying these new susceptible loci.

### Study populations

A flow chart of the study protocol was provided in Figure 1. A three-stage case-control study was designed to evaluate the association between genetic variants across human genome and the risk of CAD. To avoid the potential confounding ethnic factors and minimize sub-population stratification, cases and controls in any cohorts were recruited from the same geographical region. In stage 1 study, the “extreme cases” were defined as CAD patients with no more than one conventional risk factor. The “extreme controls” were defined as subjects with more than three risk factors but without visible coronary arterial narrowing by coronary angiography or CTA. In stage 2 and 3 studies, the subjects with >50% coronary stenosis in at least one main vessel identified by selective coronary angiography or computed tomography angiography (CTA) and those who had myocardial infarction and/or underwent percutaneous coronary intervention or coronary artery bypass graft, were classified as CAD cases. Only subjects without any history of cardiovascular diseases and without any visible coronary stenosis by coronary angiography or CTA were included as controls. The study protocol conformed to the principles of the Declaration of Helsinki and was approved by the local Ethics Committee in each hospital involving in this project. A written informed consent was given by all participants before enrollment.

### DNA extraction

Genomic DNA was extracted from EDTA-anticoagulated peripheral whole blood using the Wizard genomic DNA purification kit (Promega, Madison, MA, USA) following standard laboratory protocols.

### GWAS

In the discovery stage, genotyping was carried out in 200 “extreme individuals” (Table 1) using Infinium HumanOmnizhonghua-8 BeadChip (Illumina). High-quality genotyping was performed by a commercial company (Bioassay Laboratory of CapitalBio Corporation, Changping District, Beijing, China). Before association analysis, quality control call rates and genotyping calls for each array were analyzed by Illumina Genotyping Console software using the Dynamic Model and BRLMM-P algorithms, respectively.

### Pathway-based candidate gene case-control association study

Because of the relatively small sample size of the current GWAS, a candidate gene association study was simultaneously carried out based on known signal pathways for CAD. Based on the primary results of GWAS for CAD in the 200 “extreme individuals” in combination with earlier reports of the highthroughput dataset from a British population by WTCCC (http://www.wtccc.org.uk/), we selected 120 SNPs located in 17 chromosome regions (Chr.1, 2, 3, 4, 5, 6, 7, 8, 9, 10, 11, 12, 16, 18, 19, 20 and 22) to examine their potential associations with CAD in 293 CAD cases and sex/age matched 293 controls in Shanghai (Supplementary Table S.1).

### SNP selection and genotyping in the replication study

Only a fraction of the genotypes from the WTCCC and variants from GWAS were selected for stage 2 validation studies based on the following strict criteria: (1) SNPs had *P ≤ 10^−4^* for all GWAS samples; (2) SNPs showed consistent associations in pathway-based candidate gene case-control association study at *P ≤ 10^−2^*; (3) SNPs were not located in the same chromosome regions as reported in previous GWAS; (4) SNPs had clear genotyping clusters; (5) Only the SNP with the lowest *P* value was selected when multiple SNPs were observed in a strong linkage disequilibrium (LD) (r^2^ ≥ 0.8); (6) SNPs that had been previously reported to be strongly associated with CAD in Chinese Han population were excluded. A total of 13 SNPs representative of 7 genomic regions were selected for stage 2 validation studies and only SNPs significantly associated with CAD in stage 2 validation studies were selected for stage 3 replication studies. To determine whether common variants of *NPR-C* gene might be associated with CAD, *rs2270915 and* nineteen tag SNPs were selected with a D' > 0.9 and a r^2^ > 0.7 as a standard from the HapMap database and genotyped. In the candidate gene association study and at the validation and replication stages, genotyping was carried out with TaqMan technology (Applied Biosystems, Foster City, CA) as described previously.

### Quantitative reverse transcription polymerase chain reaction

Quantitative reverse transcription polymerase chain reaction (qRT-PCR) was performed to determine the mRNA expression of NPR-C in human peripheral blood leukocytes.

### Linkage disequilibrium (LD) and statistical genetics

Statistical analysis for GWAS was performed using previously described methods [5, 6]. With SPSS for Windows version 13.0 (SPSS Inc., Chicago, IL, USA) and PLINK software, multivariate logistic regression analysis was performed to test whether the association between a SNP and CAD remained significant after adjusting for significant risk factors for CAD. In the replication study, *P < 0.05* was deemed as statistically significant.

## SUPPLEMENTARY MATERIALS FIGURES AND TABLES




