# Supplementary material for: NPR-C gene polymorphism is associated with increased susceptibility to coronary artery disease in Chinese Han population: a multicenter study
Source: Oncotarget. 2016 May 13;7(23):33662–74. doi: 10.18632/oncotarget.9358 (PMC5085110; doi:10.18632/oncotarget.9358)
Supplement: Supplementary file 1 [file oncotarget-07-33662-s001.pdf]

NPR-C gene polymorphism is associated with increased susceptibility to coronary artery disease in Chinese Han population: a multicenter study

Supplementary Materials and Methods

Study Populations. A flow chart of the study protocol was provided in **Figure 1**. A three-stage case-control study was designed to evaluate the association between genetic variants across human genome and the risk of CAD. All study subjects were of self-described Han ethnic origin and enrolled from 5 cities or provinces: Shandong, Shaanxi, Hubei, Shanghai and Sichuan located in northern, southern and central China. To avoid the potential confounding ethnic factors and minimize sub-population stratification, cases and controls in any cohorts were recruited from the same geographical region. The population used for GWAS in stage 1 included 200 "extreme individuals" with 100 "extreme cases" and 100 "extreme controls" recruited from Shandong Province (named Dis-Shandong). The "extreme cases" were defined as CAD patients with no more than one conventional risk factor, such as age (male >45 years and female > 55 years), obesity (body mass index >26), history of cigarette smoking, hypertension, diabetes, high serum cholesterol levels, increased high sensitive C reactive protein levels (≥3mg/l), or a family history of coronary heart disease. The "extreme controls" were defined as subjects with more than three risk factors but without angiographically visible coronary arterial narrowing. The population used for candidate gene study in stage 1 involved 596 individuals (293 CAD patients and 293 controls) recruited from Shanghai (named Dis-Shanghai). The population used for SNP validation studies in stage 2 consisted of 1174 cases and

1448 controls from two independent cohorts (Val-Shandong and Val-Hubei, respectively). There were 480 cases and 469 controls in the Val-Shandong cohort and 694 cases and 979 controls in the Val-Hubei cohort. The stage 3 replication studies included 4 geographically different cohorts: Rep-Shandong, Rep-Shaanxi, Rep-Hubei and Rep-Sichuan populations, as well as two cohorts of Central Han Chinese (Rep-Center). Because Shandong and Shaanxi provinces are located in the north of China while Hubei and Sichuan provinces located in the south of China, we classified Rep-Shandong and Rep-Shaanxi as Northern Han Chinese (Rep-North), while Rep-Hubei and Rep-Sichuan as Southern Han Chinese (Rep-South). The population of Rep-Shandong and Rep-Hubei was the same as that of Val-Shandong and Val-Hubei in Stage 2, respectively. There were 392 cases and 236 controls in Rep-Shaanxi, and 480 cases and 460 controls in Rep-Sichuan. Since recent GWAS analyses have shown that the central Han Chinese can be regarded as one single homogenous population,[1] we included two cohorts of Central Han Chinese for replication studies with 293 cases and 293 controls in Cohort 1, and 1022 cases and 714 controls in Cohort 2. In an attempt to reduce the potential confounding ethnic factors, the two cohorts only included people with self-reported origin of the central Han Chinese, including indigenous people from Shanghai city, and Zhejiang, Jiangsu and Anhui provinces.

A detailed medical history was obtained from all participants by a standardized questionoaire, and a serum lipid profile (total cholesterol, HDL cholesterol, LDL cholesterol, and triglyceride levels) was obtained from the medical records. Body

mass index (BMI) was calculated as body weight (kg)/height $(m)^2$. Hypertension was diagnosed if the systolic blood pressure ≧ 140mmHg and/or diastolic blood pressure ≧ 90 mmHg or there was a history of hypertension, or the subject was taking antihypertensive drugs. Diabetes mellitus was defined as ongoing therapy for diabetes or a fasting plasma glucose level ≥7.0 mM/l. Individuals with either HDL cholesterol (HDL-C) <40 mg/dl (1.03mM/L) or LDL cholesterol (LDL-C) >160 mg/dl (4.14 mM/L) or triglycerides >200 mg/dl (2.26mM/L) were considered dyslipidemic according to Adult Treatment Panel III (ATP III) Guidelines.

All patients and control subjects underwent selective coronary angiography or computed tomography angiography (CTA) and subjects with >50% coronary stenosis in at least one main vessel identified by coronary angiography or CTA and those who had myocardial infarction and/or underwent percutaneous coronary intervention or coronary artery bypass graft, were classified as CAD cases. The diagnosis of myocardial infarction was in accordance with the international guideline published by the Joint ESC/ACCF/AHA/WHF Task Force for the Redefinition of Myocardial Infarction.[2] Subjects with coronary arterial spasm or myocardial bridge identified by coronary angiography were excluded. The control subjects were evaluated by history, physical examinations and coronary angiography or CTA. Only subjects without any history of cardiovascular diseases and without angiographically visible coronary stenosis were included as controls. Subjects with congenital heart disease, cardiomyopathy, valvular heart disease, and renal or hepatic disease were also excluded.

The study protocol conformed to the principles of the Declaration of Helsinki and was approved by the local Ethics Committee in each hospital involving in this project. A written informed consent was given by all participants before enrollment.

DNA Extraction. Venous blood was drawn from all subjects after an overnight fast. Blood, serum, and plasma were separated immediately and stored at -70°C. Genomic DNA was extracted from EDTA-anticoagulated peripheral whole blood using the Wizard genomic DNA purification kit (Promega, Madison, MA, USA) following standard laboratory protocols.

Quality Control in GWAS. In the discovery stage, genotyping was carried out using Infinium HumanOmnizhonghua-8 BeadChip (Illumina), which contained 87,2261 SNPs. High-quality genotyping was performed by a commercial company (Bioassay Laboratory of CapitalBio Corporation, Changping District, Bejing, China) following standard experimental procedures from the manufacturer (Illumina). Before association analysis, quality control call rates and genotyping calls for each array were analyzed by Illumina Genotyping Console software using the Dynamic Model and BRLMM-P algorithms, respectively. SNPs were excluded if they (i) did not map on autosomal chromosomes; (ii) had a call rate < 95%; (iii) had a minor allele frequency (MAF) < 0.05; or (iv) deviated from Hardy-Weinberg equilibrium ($P < 1 10^{-5}$) in all GWAS samples.

Pathway-based Candidate Gene Case-Control Association Study. The biological complexity of atherosclerosis implies involvement of a large number of genes and their functional variants in its pathogenesis.[3,4] Because of the relatively small sample size of the current GWAS, a candidate gene association study was simultaneously carried out based on known signal pathways for CAD. Based on the primary results of GWAS for CAD in the 200 “extreme individuals” in combination with earlier reports of the highthroughput dataset from a British population by WTCCC (http://www.wtccc.org.uk/), we selected 120 SNPs located in 17 chromosome regions (Chr.1, 2, 3, 4, 5, 6, 7, 8, 9, 10, 11, 12, 16, 18, 19, 20 and 22) to examine their potential associations with CAD in 293 CAD cases and sex/age matched 293 controls in Shanghai (**Supplementary Table S.1**).

SNP Selection and Genotyping in the Replication Study. After genome-wide association analyses, SNPs were selected for stage 2 validation studies based on the following criteria: (1) SNPs had $P \leq 10^{-4}$ for all GWAS samples; (2) SNPs showed consistent associations in pathway-based candidate gene case-control association study at $P \leq 10^{-2}$; (3) SNPs were not located in the same chromosome regions as reported in previous GWAS; (4) SNPs had clear genotyping clusters; (5) Only the SNP with the lowest *P* value was selected when multiple SNPs were observed in a strong linkage disequilibrium (LD) ($r^2 \geq 0.8$); (6) SNPs that had been previously reported to be strongly associated with CAD in Chinese Han population were excluded. These criteria were satisfied by a total of 13 SNPs (*HFE: rs2071303,*

rs2794719; CAT: rs554576, rs524154, rs7947841; HOMX1: rs2071749; CXCL9: rs2276886, rs2869460; PON3: rs2057682, rs7787187, rs11977702; LAMA4: rs6568719; NPR-C: rs700926) which are also strongly representative of the 7 genomic regions, respectively. The validation and replication studies were then conducted by two independent groups of investigators, one in Shandong University and another in the Chinese National Human Genome Center in Shanghai. Only SNPs that showed significant associations with CAD in stage 2 validation studies were selected for stage 3 replication studies.

To determine whether common variants of the *NPR-C* gene might be associated with CAD, the Shandong research group firstly genotyped nine tag SNPs of NPR-C *(rs1833529, rs2270915, rs17541471, rs3792758, rs696831, rs7715279, rs6450922, rs10941022 and rs976576)* selected from the HapMap database. The Shanghai research group then genotyped *rs2270915* and additional ten tag SNPs (*rs9716700, rs11750438, rs6889608, rs10036648, rs12697273, rs10066436, rs3828586, rs10061804, rs2062708 and rs7730564*). Moreover, the linkage disequilibrium (LD) of these tag SNPs with other SNPs were assessed respectively using the data from HapMap by D' and r^2, and the results showed that the common variations could be tagged almost by these 20 chosen tag SNPs with a D' > 0.9 and an $r^2 > 0.7$ as standard (**Supplementary Figure S.4**). Thus, these tag SNPs had relatively strong representativeness for *NPR-C* SNPs.

In the candidate gene association study and at the validation and replication stages, genotyping was carried out with TaqMan technology (Applied Biosystems, Foster

City, CA) as described previously.[5] TaqMan genotyping assays with probes labeled with the fluorophores FAM and VIC were purchased or ordered from Applied Biosystems. Genotyping was performed on 96-well plates using an ABI PRISM® 7500 Real-Time PCR instrument. The PCR conditions were 1 cycle at 95°C for 10 min, followed by 40 cycles of 92°C for 15 sec and 60°C for 1 min as recommended by the manufacturer. The Universal PCR Master Mix from Applied Biosystems was used in a 7.5μL total reaction volume with 10 ng DNA per reaction. Allelic discrimination was measured with Sequence Detection Systems 1.4 software. A series of methods was used to control the quality of genotyping: (1) case and control samples were mixed on each plate and genotyped without the knowledge of their clinical data; (2) two water controls in each plate were used as blank controls; (3) 5% of the samples were randomly selected to repeat the genotyping, as blind duplicates, and the reproducibility was 100%; (4) to verify the genotyping results by TaqMan assays, we randomly selected 96 DNA samples and genotyped rs700926 by direct DNA sequence analysis. The genotyping data from the TaqMan assays agreed completely with that obtained through DNA sequencing with a concordance rate of 100%.

Quantitative Reverse Transcription Polymerase Chain Reaction. Venous blood was drawn from all subjects after an overnight fast. Total RNA was extracted from human peripheral blood leukocytes using TRIzol reagent (Invitrogen, Karlsruhe, Germany) following the manufacturer’s instruction. The mRNA expression levels of

NPR-C and β-actin in leukocytes were determined by means of SYBR Green technology (Applied Biosystems). The housekeeping gene β-actin was quantified as an internal RNA control. Amplification was performed with 40 cycles and annealing at 55-58°C for 30 sec and at 72 °C for 35 sec extension with an Applied Lightcycler 2.0 detection system (Roche Applied Science, USA) according to the manufacturer's instruction. Quantitative reverse transcription polymerase chain reaction (qRT-PCR) was performed to determine the mRNA expression of NPR-C in leukocytes. All real-time PCR reactions, including no-template controls and real-time minus controls, were performed in triplicate using the primers as follows: NPR-C- forward: CGCATTTCAAAACGACCTTCTT, NPR-C- reverse: CCGGGCAGGTGTCCATAG; GAPDH-forward: 5′-GAAGGTGAAGGTCGGAGT-3′, GAPDH-reverse: 5′-GAAGATGGTGATGGGATTTC-3′. GAPDH was used as a reference gene. A relative expression was calculated using the equation $2^{-\Delta Ct}$ (Ct, Cycle Threshold), where $\Delta Ct = Ct_{Target\ gene} - Ct_{GAPDH}$.

Because rs700926 is located near intron 1 of NPR-C, we hypothesized that it may affect the expression levels of the NPR-C mRNA. Thus, we randomly selected 5 individuals with a minor genotype of GG, 27 individuals with genotype GT and 63 individuals with genotype TT of rs700926 for RT-PCR analysis. To validate the finding of a quantitative expression trait locus from the initial analysis, we performed a replication study in an independent population of 380 randomly selected individuals from the replication populations (northern and southern Chinese) as described above.

Linkage Disequilibrium (LD) and Statistical Genetics. The statistical analysis methodology of the GWAS was described previously.[6] LD between the SNPs was estimated by the expectation maximization (EM) algorithm using Arlequin 2.0. Deviations from Hardy-Weinberg equilibrium were assessed by χ^2 test using Haploview 4.0 (http://www.broad.mit.edu/mpg/haploview/). *P* values and corresponding Odds Ratios (ORs) and 95% confidence intervals (CIs) were computed for each SNP. We expressed the continuous variables as mean ± SD and determined variance equality by the Bartlett's test. Univariate analysis used to measure the association of each SNP with CAD was tested by χ^2 test. Clinical and anthropometric characteristics were compared between CAD patients and controls by analysis of variance (ANOVA) or logistic regression. Covariable adjustments were carried out as indicated. Multivariate logistic regression analysis was performed to test whether significant association between a SNP and CAD remained after adjusting for significant risk factors for CAD (age, gender, smoking, hypertension, diabetes and serum lipid levels). R software (version 2.11.1; The R Foundation for Statistical Computing, http://www.cran.r-project.org/) was also used for statistical analysis and generating plots, including Q-Q plot and Manhattan plot. The SPSS for Windows version 13.0 (SPSS Inc., Chicago, IL, USA) and PLINK (v1.07, http://pngu.mgh.harvard.edu/~purcell/plink/) software were applied to undertake statistical analysis. Statistical power and sample size determination for validation and replication populations were estimated using a free Power and Sample size calculation program (PS version 3.0.43). In replication study, the criterion for statistical

significance was $P < 0.05$.

References

1. Chen J, et al. Genetic structure of the Han Chinese population revealed by genome-wide SNP variation. Am J Hum Genet. 2009; 85: 775-785.

2. Thygesen K, et al. Universal definition of myocardial infarction. Circulation. 2007; 116: 2634-53

3. Kullo IJ, Ding K. Mechanisms of disease: The genetic basis of coronary heart disease. Nat Clin Pract Cardiovasc Med. 2007; 4: 558-69.

4. Lutucuta S, Ballantyne CM, Elghannam H, Gotto AM Jr, Marian AJ. Novel polymorphisms in promoter region of atp binding cassette transporter gene and plasma lipids, severity, progression, and regression of coronary atherosclerosis and response to therapy. Cir Res. 2001; 88: 969-73.

5. Shen GQ, Abdullah KG, Wang QK. The TaqMan method for SNP genotyping. Methods Mol Biol. 2009; 578: 293-306.

6. Samani NJ, et al. Genomewide association analysis of coronary artery disease. N Engl J Med. 2007; 357: 443-53.

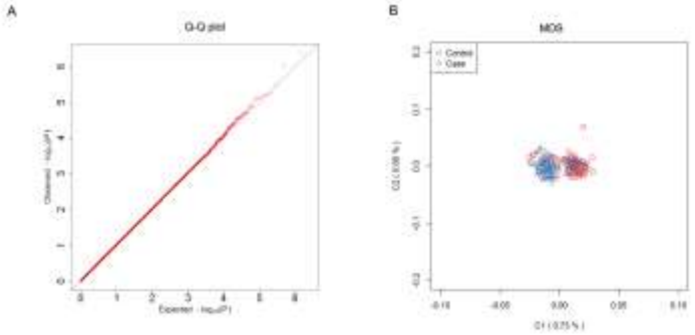

Figure S.1 (A) Quantile-Quantile (Q-Q) plots of the observed *P* values for association in initial GWAS. (B) Multidimensional scaling (MDS) was used to exclude population outliers.

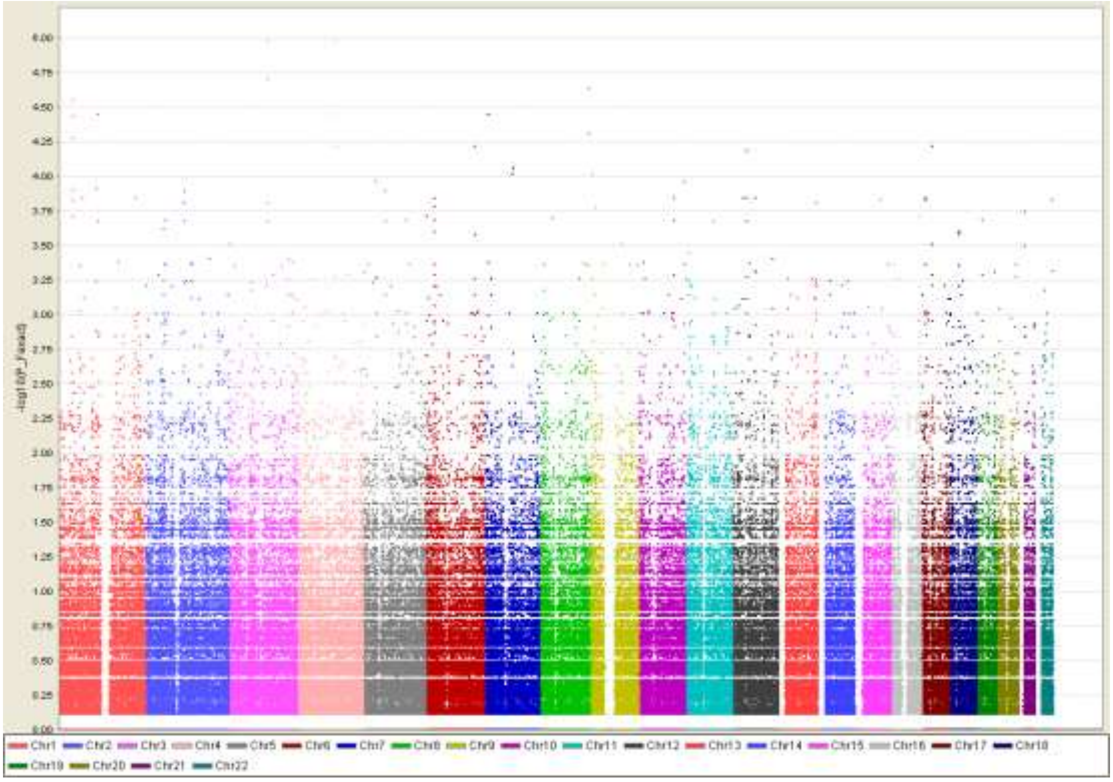

Figure S.2 Plot of -log10(*P*) for association of single-nucleotide polymorphisms and chromosomal position for all autosomal single-nucleotide polymorphisms analyzed in the age- and sex adjusted model of coronary artery disease in Dis-Shandong cohort.

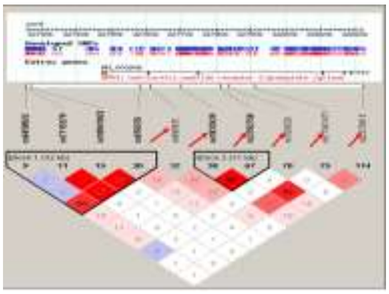

Figure S.3 Block structure of linkage disequilibrium for the 10 selected tagging SNPs (*rs700926, rs1833529,rs2270915, rs17541471, rs3792758, rs696831, rs7715279, rs6450922, rs10941022 and rs976576*) in the *natriuretic peptide receptor C* gene region (*NPR-C*) on chromosome 5p13.3.

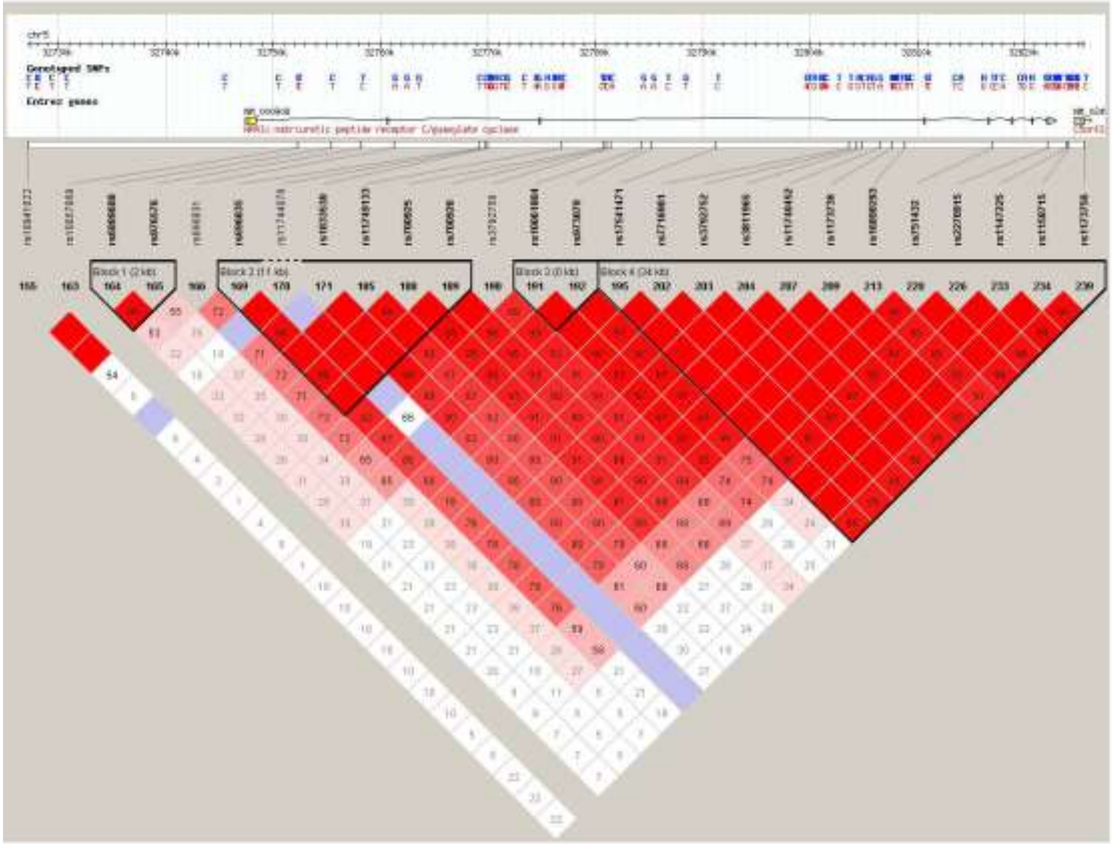

Figure S.4 Block structure of linkage disequilibrium for all SNPs in the *natriuretic peptide receptor C* gene region (*NPR-C*) on chromosome 5p13.

Table S.1 Summary of the associations between 120 SNPs and CAD risk in signal pathway-based candidate gene study in Dis-Shanghai cohort

For Table S1, please see the attached Excel file

Table S. 2 GWAS scan for association between SNPs of *NPR-C, HMOX-1, CAT, CXCL6, HFE, LAMA4* and *PON3* and risk of CAD in Dis-Shandong cohort.

For Table S2, please see the attached Excel file

Table S.3 Allelic association of SNPs of *HFE*, *CAT*, *HMOX1*, *CXCL9*, *PON3*, and *LAMA4* with CAD in the Val-Shandong and Val-Hubei cohorts.

Gene	SNP_ID	Minor Allele	MAF(%)*		OR (95%CI)†	*P-obs*‡
			Case	Control		
**HFE**	rs2071303	T	29.3	30.7	0.94(0.80-1.10)	0.410
	rs2794719	T	30.5	30.9	0.98(0.84-1.15)	0.800
**CAT, ELF5**	rs554576		34.8	36.2	0.94(0.81-1.10)	0.440
	rs524154	T	31.5	31.1	1.02(0.87-1.19)	0.810
	rs7947841	A	32.1	34.9	0.88(0.75-1.03)	0.1000
**HMOX1**	rs2071746	T	44.4	44.0	0.99(0.82-1.18)	0.620
**CXCL9**	rs2276886	A	42.6	41.0	1.07(0.94-1.21)	0.287
	rs2869460	G	47.2	47.5	0.99(0.87-1.11)	0.839
**PON3,**	rs2057682	C	19.7	18.2	1.10(0.94-1.29)	0.239
	rs7787187	T	2.67	2.58	1.03(0.7-1.53)	0.873
**PON3,PON2**	rs11977702	T	18.1	16.8	1.09(0.92-1.29)	0.304
**LAMA4**	rs6568719	C	38.02	38.15	0.99(0.84-1.18)	0.948

*MAF: minor allele frequency. †OR, odds ratio, CI, confidence interval. ‡*P-obs*, uncorrected *P* value.
